# Elevated tacrolimus exposure variability predicts transplant renal insufficiency

**DOI:** 10.3389/fimmu.2026.1656392

**Published:** 2026-02-13

**Authors:** Yue Zhao, Ying-Xin Zhao, Di Zhao, Wei-Li Wang, Qian Wang

**Affiliations:** 1 Department of Pharmacy, the First Affiliated Hospital of Army Medical University (Third Military Medical University), Chongqing, China; 2 Department of Pharmacy, Daping Hospital, Army Medical University (Third Military Medical University), Chongqing, China; 3 Department of Nephrology, the First Affiliated Hospital of Army Medical University (Third Military Medical University), Chongqing, China

**Keywords:** coefficient of variation, prediction, tacrolimus, time in therapeutic range, transplant renal insufficiency

## Abstract

**Background:**

Optimizing immunosuppression based on immunological risk profiles remains the preferred regimen for improving long-term graft outcomes after renal transplantation. Tacrolimus (TAC), the cornerstone drug of the calcineurin inhibitors, is currently the most commonly used immunosuppressant for renal transplantation. Under-exposure can lead to rejection events, while overexposure has been shown to cause drug-related renal injury. This study aims to assess the impact of TAC exposure on graft renal function to identify high-risk individuals of post-transplantation.

**Methods:**

The present study employed a single-center retrospective cohort design to enroll adult recipients who underwent allogeneic kidney transplantation from 1 January 2019 to 31 December 2023. Cox regression analysis was used to analyze the independent risk effects of exposure parameters, which included mean, median, standard deviation (STD) and coefficient of variation (CV). Hereafter, the Kaplan-Meier method was applied to perform the survival analysis of stabilized graft renal function.

**Results:**

69 cases (19.22%) in the study cohort developed graft renal insufficiency. And a STD of TAC target trough concentration≥5 was associated with a 4.55-fold elevated risk (95% CI 1.60-12.95, p = 0.004). Recipients in the higher CV had an increased risk of graft renal insufficiency in comparison with those in low CV tertile. As indicated by the survival analysis curves, it was determined that TAC mean trough concentrations >8 ng/mL, CV>0.5 and TTR<0.3 were associated with a higher risk of graft renal insufficiency.

**Conclusion:**

Higher TAC trough concentrations, higher CV, and lower TTR post-transplantation significantly increases the risk of transplant renal insufficiency. Identifying these high-risk transplant recipients, correcting potentially influential factors and keeping TAC within recommended concentrations will significantly improve the outcomes and survival of renal transplant recipients.

## 1 Introduction

Renal transplantation has become the most effective measure to save the lives of patients with chronic renal failure and also is the best alternative treatment for patients with end-stage renal disease (1–3). The success of renal transplantation relies heavily on immunosuppressive therapy, particularly calcineurin inhibitors, such as tacrolimus (TAC) and cyclosporine A (4, 5). These drugs are essential for reducing rejection and improving the long-term outcomes after transplantation. TAC has been shown to have a stronger immunosuppressive effect than cyclosporine A with a reduction in acute rejection by at least 30% and having a higher long-term graft renal survival rate (6, 7). In addition, TAC has been demonstrated to have a lower incidence of nephrotoxicity and is less likely to cause hyperlipidemia and hypercholesterolemia (8). Consequently, TAC has been recommended as the first-line immunosuppressive drug in calcineurin inhibitors following renal transplantation. However, it is characterized by a narrow therapeutic window, meaning that drug concentrations must be tightly controlled in clinical applications. Under-exposure can lead to rejection events, while overexposure has been shown to cause drug-related renal injury (9, 10). Irrational use of TAC can impair graft function and compromise the long-term survival of both the graft and recipient. Therefore, routine therapeutic drug monitoring (TDM) of its trough concentration levels can optimize dosing decisions and ensure these levels remain within the desired target range.

Conventional single-point trough level monitoring has a limited capacity to capture and manage the high intrapatient variability (IPV) in TAC pharmacokinetics. This IPV is often driven by modifiable factors such as medication non-adherence, drug-drug interactions, and post-transplant gastrointestinal complications (11). Consequently, many investigators have initiated the exploration of enhanced exposure parameters that are indicative of renal transplantation outcomes. TAC immunosuppressive variability measures the fluctuation of trough concentrations in a patient over a specific time period. This variability is quantified by various metrics such as standard deviation (STD) and coefficient of variation (CV) (12, 13). Previous studies have shown that TAC immunosuppression variability has been recognized as a new marker for assessing the risk of adverse outcomes in renal transplantation, including graft rejection, graft loss and graft renal insufficiency (14, 15). These results are not entirely consistent across the different coefficient of variation indices. The specific differences between these coefficients of variation metrics, the criteria for determining thresholds, and the impact of the timing of calculations on clinical outcomes are currently under-explored. In addition, previous studies have generally considered TAC trough levels in the range of 5–10 ng/ml to be the effective therapeutic concentration (16). However, the effective concentration range of TAC varies at different stages post-transplantation. China updated the TAC concentration monitoring range in 2024 based on the 2016 Guidelines for Immunosuppressive Therapy in Chinese Kidney Transplant Recipients. And no study has yet explored the correlation between TAC concentrations and the risk of transplant renal insufficiency at different times after transplantation (17).

This study expects to describe the role of TAC in preventing graft renal insufficiency in renal transplant patients receiving triple immunosuppressive therapy at a single center. We firstly evaluate the relationship between five different TAC exposure parameters and the risk of transplant renal insufficiency during four-time interval after renal transplantation. Cox’s proportional hazards regression model was used to assess the hazard ratios of transplant renal insufficiency. Finally, Kaplan-Meier curves were calculated for the five TAC exposure parameters. The objective of the study was to identify those at high-risk of graft failure and to improve long-term graft survival and clinical prognosis in renal transplant recipients by optimizing the management of TAC therapeutic window concentrations.

## 2 Materials and methods

### 2.1 Study population and data collection

This retrospective study included recipients who received allogeneic renal transplantation at the First Affiliated Hospital of Army Medical University between January 2019 to December 2023. The inclusion criteria were as follows: (i) patients >18 years, (ii) those who received a triple immunosuppressive regimen consisting of TAC, mycophenolate mofetil (or mycophenolic acid), and glucocorticoids, (iii) post-transplantation follow-up of at least 6 months, and (iv) at least six TAC trough measurements at the outpatient clinic. We excluded patients who (i) lacked sufficient trough concentrations, (ii) had missing serum creatinine (Scr) measurements, (iii) elevated creatinine or death due to infection, obstruction, etc.

The following demographic and clinical information were collected: age, sex, pre-transplant renal primary disease, induction protocol, donor kidney source, renal pathology biopsy result, warm ischaemia time (WIT) and cold ischaemia time (CIT). And other laboratory dada, such as TAC trough levels, Scr were extracted from the electronic medical records. Recipients were strictly divided into transplant renal insufficiency and stable transplant renal function groups based on postoperative renal function during the follow-up period. The transplant renal insufficiency was determined by one of these criteria: (i) the presence of rejection of the transplant kidney confirmed by biopsy, (ii) increased Scr level: Scr level reaching twice the level of 3 months after transplantation and lasting for more than 14 days, and (iii) the loss of the graft, the recipient resuming long-term dialysis, or the death of the transplanted kidney due to dysfunction. Ethical approval [(B)KY2025067] was granted by the Ethics Committee of the First Affiliated Hospital of Army Medical University prior to the commencement of this study, and the requirement for informed consent was removed for a retrospective observational study.

### 2.2 Immunosuppression regimen

The immunosuppression regimen was performed according to center protocol. The induction therapy includes Rabbit anti-thymocyte globulin (ATG, thymoglobulin, 1 mg/kg administered for 3–7 days) or basiliximab (20 mg on days 0 and 4 post-transplant). The selection of induction regimens is primarily based on patient immune risk. Recipients who are PRA-negative and have a low HLA mismatch burden are generally considered low immune risk, while those with ABO blood type incompatibility or DSA positivity are classified as high immune risk. ATG is selected as the induction agent for high immune risk recipients. Maintenance immunosuppression was TAC, mycophenolate mofetil or enteric-coated mycophenolate sodium (EC-MPS) and steroids. The initial dose of tacrolimus is 0.05–0.15 mg/kg/day. For MMF, the recommended dose is 0.5–1.0 g q12h, and for EC-MPS, the recommended dose is 540–720 mg q12h. The standard induction regimen involves intravenous administration of methylprednisolone 500–1,000 mg (10–15 mg/kg) during transplantation. Starting on postoperative day 4, prednisolone is switched to oral administration, beginning at 10–30 mg/day. By postoperative day 30, the dose is gradually tapered to a maintenance level of 10–15 mg/day. Upon entering the maintenance phase, most transplant centers employ low-dose maintenance therapy: 10 mg/day at 2–3 months, 5–10 mg/day at 6 months, and 5–10 mg/day thereafter. TAC trough levels were obtained at 8:00-10:00 a.m. on each occasion and measured by the enzyme multiplied immunoassay technique (EMIT) each time prior to the administration of the drug (18). It is recommended that within 2 weeks after transplantation, monitoring should be performed twice weekly until the target concentration is reached. In general, patients were advised to receive routine follow-up weekly during 1 to 2 times weekly for 3 to 4 weeks after surgery, 1 time weekly for 1 to 3 months after surgery, every 3 to 4 weeks for 4 to 12 months after surgery, every 1 to 2 months for more than 1 year after surgery, and at least once every quarter for more than 5 years thereafter.

### 2.3 Calculation of TAC intra-patient variability

TAC blood concentration monitoring data were extracted from the electronic medical record system to focus on the analysis of TAC intra-patient variability at four-time interval, including within 1 month, 1–3 months, 3–12 months, and >12 months postoperatively. After collecting all data, we assess these points based on clinical symptoms and other laboratory test indicators. Concentration points that are extremely low (<2 ng/mL) or extremely high (>20 ng/mL) and cannot be explained by clinical circumstances, such as an acute infection or a medication error, are considered outliers. The minimum number of TAC measurements monitored at each window for each sample must be >3 times. In this study, TAC exposure was characterized by its central tendency (mean and median trough levels) and its intra-patient variability. The latter was primarily assessed using the coefficient of variation (CV) which is the standard metric for IPV. The standard deviation (STD) is also reported as an absolute measure of dispersion. Time in therapeutic range (TTR), which calculates the percentage of time a patient is within a predefined target range, has emerged as the standard to assess effective warfarin management in both clinical and research settings. According to the 2023 Chinese Guidelines for Immunosuppressive Therapy in Renal Transplant Recipients, TAC concentrations are recommended as follows, with a trough concentration range of 8–12 ng/mL for within 1 month, 6–10 ng/mL for 1–3 months, 5–10 ng/mL for 4–12 months, and 5–8 ng/mL for >12 months (17).


$$\text{CV}=\frac{\text{STD}}{\text{MEAN}}$$

### 2.4 Statistical methods

Continuous variables were expressed as the mean ± STD and categorical variables were described as relative frequencies. Categorical variables were compared using the χ^2^ test or Fisher’s exact test. A student t-tests were used to assess continuous variables. The Cox proportional hazards regression model was used to obtain estimates of hazard ratios for transplant renal insufficiency and p<0.05 was considered statistically significant. In multivariate analysis, we assess multicollinearity by calculating variance inflation factors (VIFs). For all continuous variables and dummy variables derived from categorical variables, we examine VIF after model fitting. Typically, VIF > 5 indicates moderate multicollinearity, while VIF > 10 signifies severe multicollinearity. If severe multicollinearity is detected, we assess the clinical importance of the relevant variables and consider removing one or combining them into a composite indicator. Kaplan-Meier probabilities of transplant renal insufficiency survival were plotted and compared by TAC levels using log rank tests. All statistics were performed using the statistical software Python version 3.9.

### 2.5 The methodology of research

When calculating the Cox proportional hazards regression model, TAC exposure parameters within each time window must be calculated individually, and and endpoint events occurring within that window are calculated separately. If an endpoint event occurs, the patient will be excluded from subsequent analyses. For example, if patient A experiences graft dysfunction at 3 months post-transplant, only the HR for TAC concentration associated with graft dysfunction during the 1–3 months period is assessed, and this patient is excluded from subsequent phase analyses. However, in KM curve analysis, we do not differentiate monitoring periods, and all monitoring point data are directly incorporated into the analysis until the endpoint event occurs. For example, the KM curve of patient A will utilize all TAC concentration monitoring data from the 0–3 months post-transplant period.

## 3 Results

### 3.1 Baseline characteristics

The study included 359 patients (30.47% women), who were a median transplant age of 35 (IQR, 27–43) years. Baseline characteristics for all patients are shown in **Table 1**. The majority of donors were from donation after cardiac death (DCD), and indications for transplantation include hypertensive nephropathy (35.46%), IgA nephropathy (15.60%), and in many cases, chronic renal failure (30.92%) that has been diagnosed at the time of the initial visit. At census, 52 patients (14.4%) had rejection and 17 (4.43%) had developed chronic kidney allograft dysfunction, including creatinine elevation, renal allograft dysfunction and graft nephrectomy. The dynamic exposure parameters of tacrolimus were statistically significant in the group with stable renal function and in the group with renal insufficiency, while the other parameters did not demonstrate statistical significance.

**Table 1:** Baseline characteristics of patients.

Variables	Total cohort (n=359)		Stable renal function group (n=290)	Renal insufficiency group (n=69)	*p*
**Famale**	109 (30.47%)		91 (31.38%)	18(26.09%)	0.390
**Transplant age**	35 (27-43)		35 (27-43)	34(28-40)	0.467
Transplant indication					
Hypertensive renal disease	128(35.46%)		100(34.48%)	28(40.58%)	0.126
IgA nephropathy	56(15.60%)		42(14.48%)	14(20.29%)	
Unknown reason	111(30.92%)		96(33.10%)	15(21.74%)	
ADPKD	13(3.62%)		8(2.76%)	5(7.25%)	
Donor type					
DCD	272 (75.77%)		217 (74.83%)	53 (76.81%)	0.732
Living	89 (24.79%)		73 (25.17%)	16 (23.19%)	
HLA					
**<3**	142 (39.55%)	111 (38.28%)		31 (44.92%)	0.014
**≥3**	217 (60.44%)	179 (61.72%)		38 (55.07%)	0.380
DSA					
**1**	13 (3.62%)	12 (4.14%)		1(1.45%)	0.476
**0**	346 (96.38%)	278 (95.86%)		68(98.55%)	
Induction agent,n (%)					
Basiliximab	170 (47.35%)		148(51.03%)	22(31.88%)	0.210
Anti-thymocyte globulin	189 (52.65%)		142(48.97%)	47(68.12%)	
**Warm ischemia time, min**	3.7 (3-5)		3.7 (3-5)	3.70 (2-5)	0.817
**Cold ischemia time, h**	3.4 (1.5-4)		3.3(1.5-4)	3.62 (1.5-5)	0.378
**Median of TAC, ng/ml**	6.70 (5.35-8.25)		6.70 (5.40-8.30)	6.00 (4.70-7.00)	**0.003**
**Mean of TAC, ng/ml**	7.00 (5.63-8.59)		7.02 (5.64-8.62)	6.57 (4.88-7.73)	0.056
**STD of TAC**	1.80 (1.15 -2.80)		1.78 (1.14-2.73)	2.14 (1.29-3.29)	0.101
**CV of TAC**	0.27 (0.19-0.38)		0.27 (0.19-0.38)	0.36 (0.24-0.52)	**0.002**
**TTR of TAC**	0.46 (0.15-0.75)		0.46 (0.15-0.76)	0.40 (0.12-0.68)	0.509
**Total follow-up time, months**	28 (16-45)		32 (18-48)	23 (5-52)	**<0.001**

ADPKD, autosomal dominant polycystic kidney disease; DCD, donation after cardiac death; HLA, human leukocyte antigen; DSA, donor specific antibody; TAC, tacrolimus; STD, standard deviation; CV, coefficient of variation; TTR, time in therapeutic range.

Bold values indicate statistical significance.

### 3.2 Correlations among metrics of TAC trough levels

**Figure 1:**
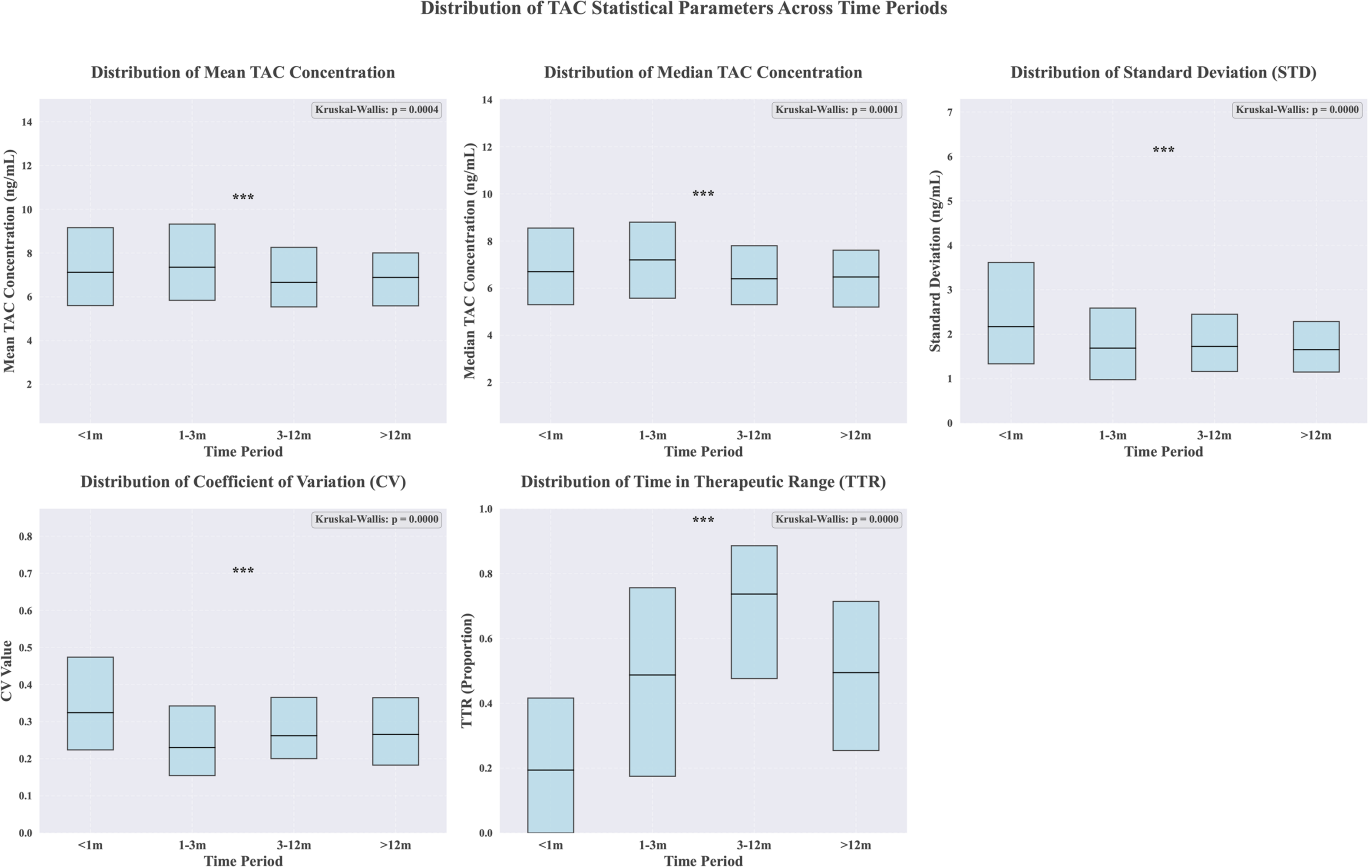
Mean, median, STD, CV and TTR of TAC trough levels at various times after transplantation. *** means p< 0.001.

The median of TAC trough level was 6.70 (IQR, 5.15–8.65) ng/ml, 7.20 (IQR, 5.60–9.00) ng/ml, 6.45 (IQR, 5.35–7.90) ng/ml and 6.40 (IQR, 5.28–7.60) ng/ml for the epochs 0 to1 months, 1 to 3 months, 3 to 12 months, and >12 months, respectively. And TAC TTR values in the four time periods were all statistically significant, with distributions of 0.19 (IQR, 0-0.42) ng/ml, 0.49 (IQR, 0.17-0.76) ng/ml, 0.74 (IQR, 0.48-0.88) ng/ml and 0.49 (IQR, 0.25-0.71) ng/ml, respectively (**Figure 1**).

**Figure 2:**
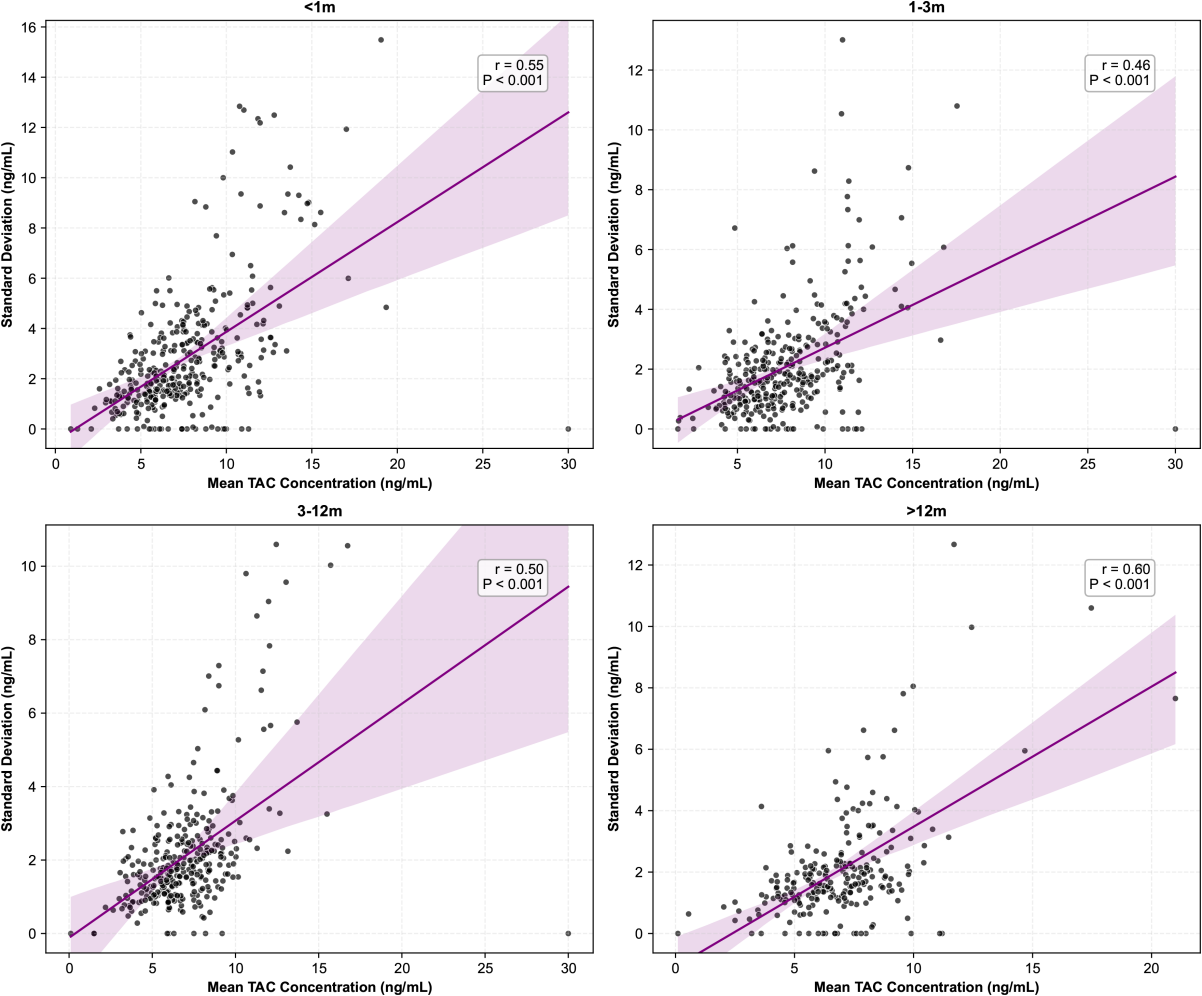
STD vs mean TAC level at various times after transplantation.

**Figure 3:**
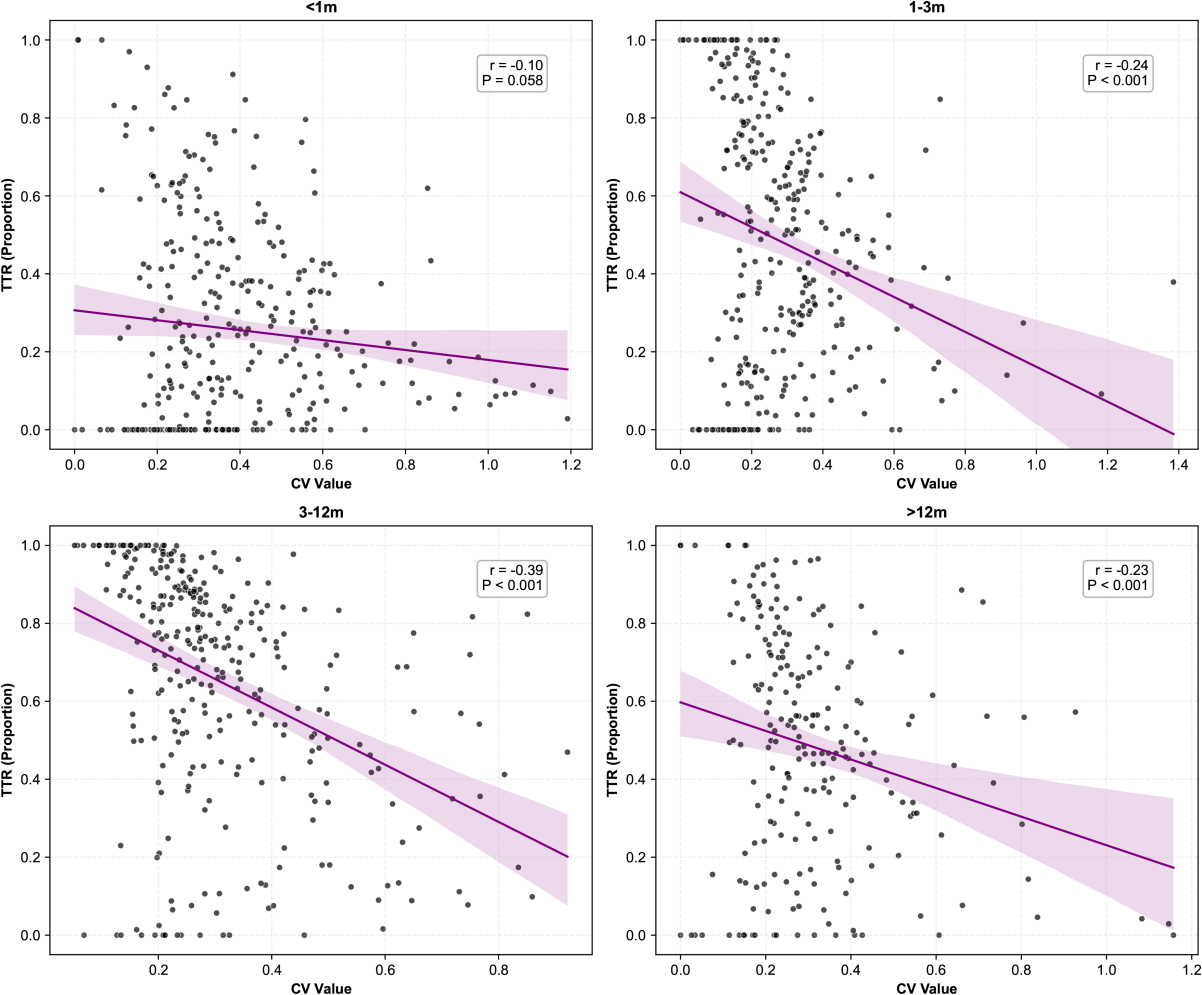
TTR vs CV TAC level at various times after transplantation.

The STD TAC level showed a strong positive correlation with mean TAC C0 range over 4 different period, with r ranging from 0.46 to 0.60 (p<0.001, **Figure 2**). In addition, there is a similar correlation was observed for the median and standard deviation values of tacrolimus concentrations (**Supplementary Figure S1**). Conversely, the CV and TTR values for tacrolimus concentration showed a negative correlation, except during the first month post-transplantation (p<0.001, **Figure 3**).

### 3.3 Association between multiple TAC exposure parameters and the risk of graft renal insufficiency

**Figure 4:**
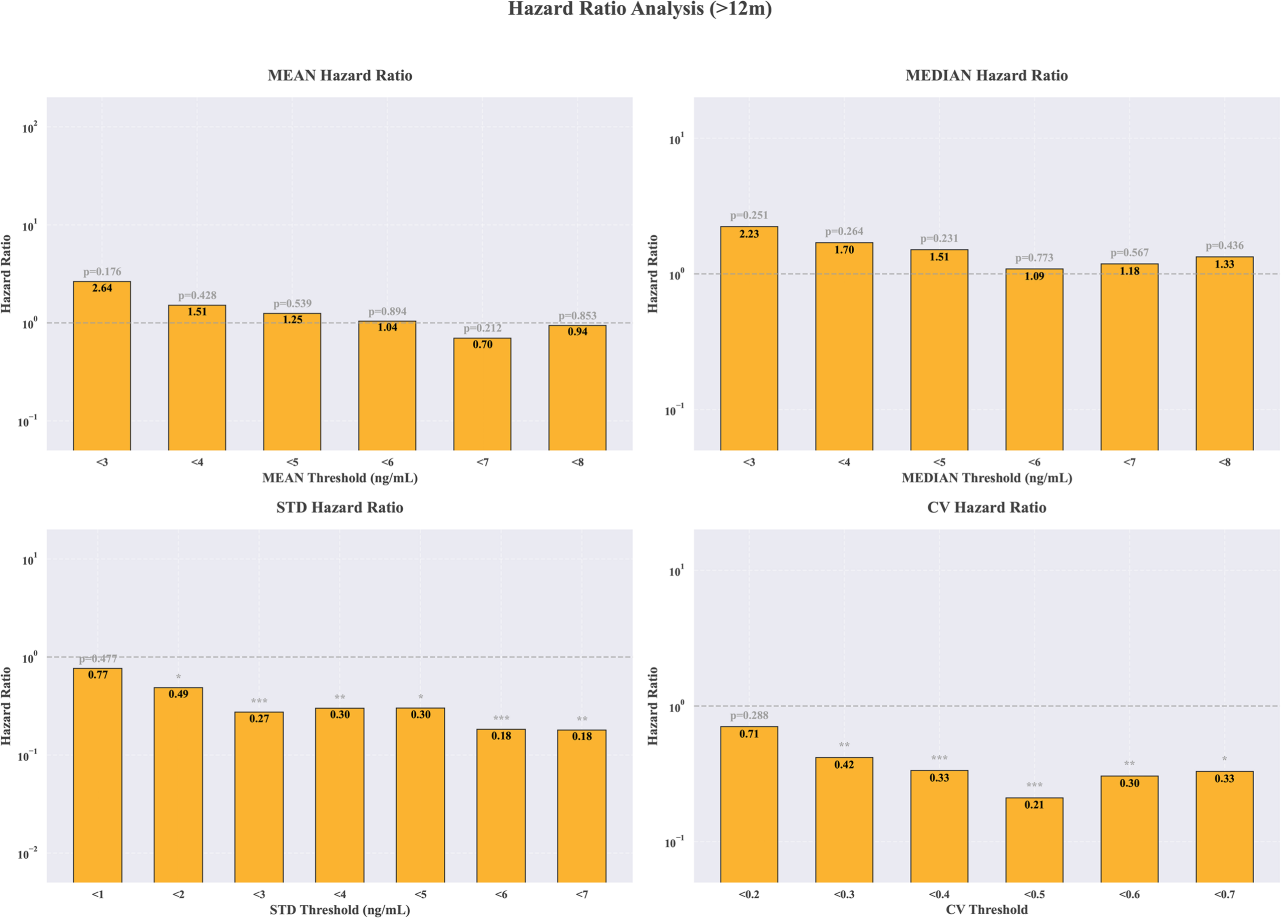
Hazard ratio for graft renal insufficiency using a binary cutoff of ≥ or < mean, median, STD and CV of TAC level.

Firstly, an investigation was conducted into the HR of differing exposure parameters to transplant renal insufficiency by refining multiple thresholds. **Figure 4** shows graft renal insufficiency risk associated with TAC trough level at different cut-points for 1 months, 1–3 months, 3–12 months and >12 months. Since only one case of graft dysfunction occurred one month postoperatively and five cases occurred between 1–3 months postoperatively, the HR for these two time periods will not be discussed further. In the period of >12 month following transplantation, the likelihood of graft renal insufficiency was 79% lower in the group of CV<0.5, in comparison to the group of CV> 0.5 (p < 0.001). Furthermore, it has been demonstrated that a lower STD value is associated with a lower risk of transplant renal insufficiency (HR 0.27 95% CI 0.14-0.52, p<0.001). The HR values for tacrolimus concentrations between 3–12 months post-transplant and transplant renal insufficiency were not statistically significant (**Supplementary Figure S2**).

**Figure 5:**
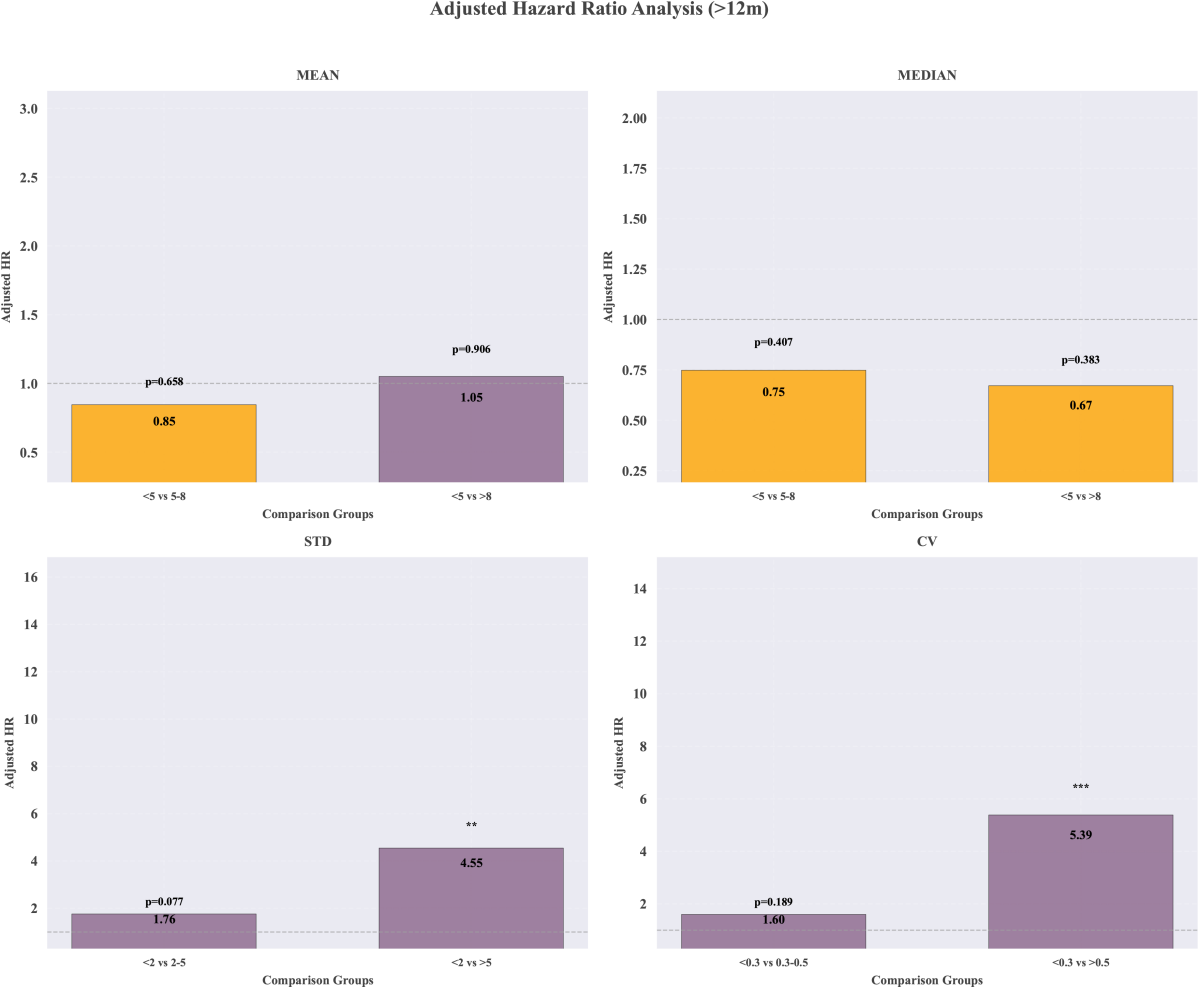
Adjusted hazard ratios of graft renal insufficiency by TAC level metrics (mean, median, STD, CV).

**Figure 6:**
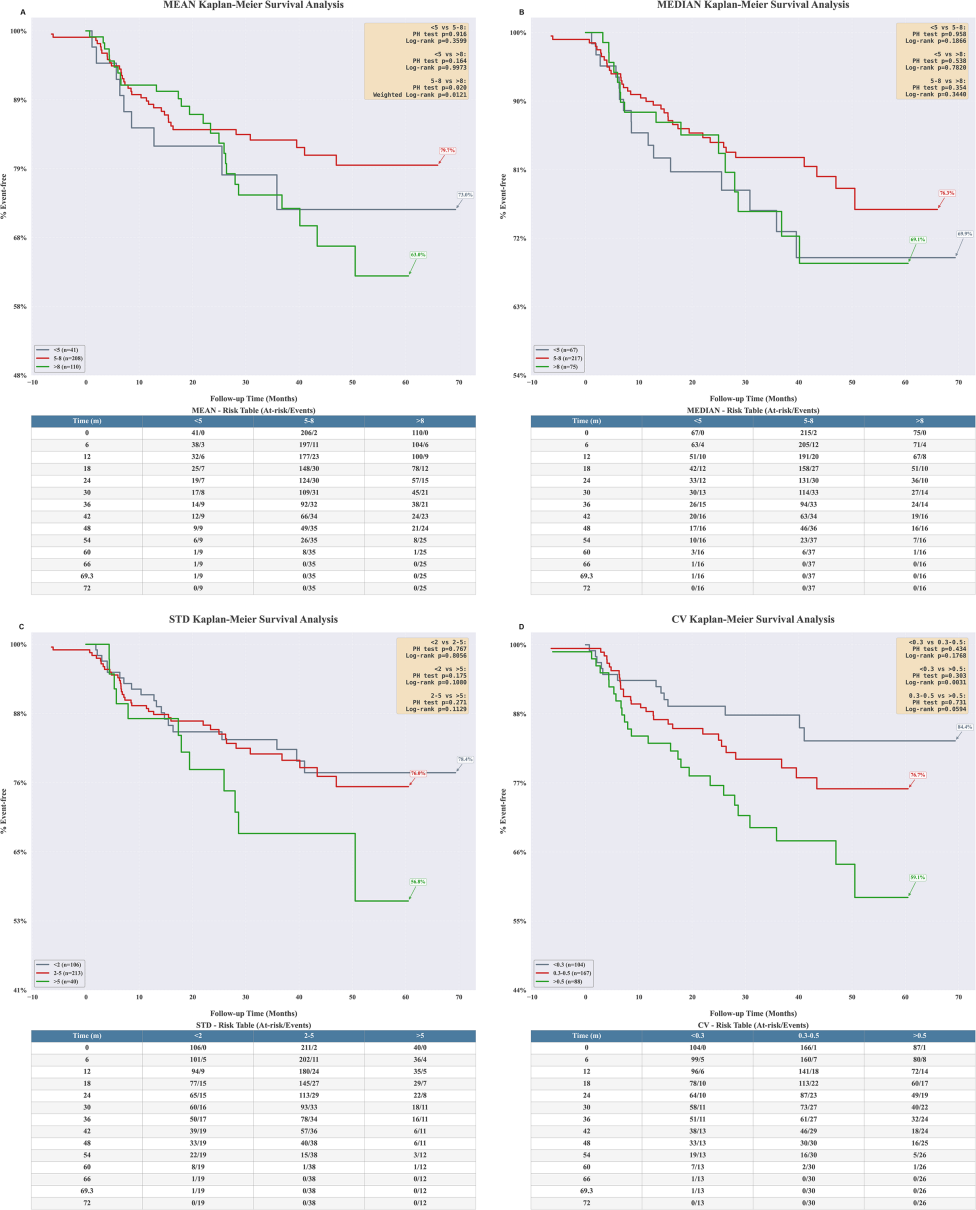
Kaplan-Meier plots for percent graft renal insufficiency-free survival.

Based on above risk thresholds, the HR values were recalculated by refining multiple thresholds shown in **Figure 5** and **Table 2**. The results from subgroup analyses were consistent. Both univariate and multivariate-adjusted analyses demonstrated that elevated CV levels (CV ≥ 0.5) and elevated STD levels (STD ≥ 5) were associated with a higher risk of transplant renal insufficiency, with HR of 5.39 and 4.55, respectively. In contrast, neither the median nor the mean tacrolimus concentration showed statistical significance. Meanwhile, the exposure index of TAC at 3–12 months did not significantly suggest a significant association with transplant renal insufficiency (**Supplementary Figure S3**). For the median TAC concentration results, we observed a significant survival difference between patients maintaining concentrations consistently between 5–8 ng/mL and those in the high-concentration group (≥8 ng/mL). A coefficient of variation (CV) ≥0.5 emerged as a key predictor of poor prognosis, with these patients demonstrating significantly lower graft function stability rates compared to those with a low CV (<0.3), as shown in **Figure 6**.

**Table 2:** Univariate and multivariable analysis (adjusted for age, sex, ethnicity, history of prior transplant, donor type, HLA match and induction therapy) for tacrolimus at 12 months and risk of graft renal insufficiency.

Tac exposure parameters			HR (95% CI)	P-value
CV	<0.3 vs. 0.3-0.5	Univariate	1.60 (0.79-3.22)	0.189
		Multivariable	1.60 (0.79-3.23)	0.188
	<0.3 vs. ≥0.5	Univariate	5.27 (2.45-11.36)	**<0.001**
		Multivariable	5.39 (2.48-11.69)	**<0.001**
TTR	<0.3 vs. 0.3-0.5	Univariate	0.83 (0.39-1.75)	1.754
		Multivariable	0.96 (0.44-2.08)	0.915
	<0.3 vs. ≥0.5	Univariate	0.66 (0.33-1.31)	0.233
		Multivariable	0.66 (0.33-1.32)	0.240
STD	<2 vs. 2-5	Univariate	1.78 (0.96-3.23)	0.069
		Multivariable	1.76 (0.94-3.30)	0.077
	<2 vs. ≥5	Univariate	4.27 (1.52-11.97)	**0.006**
		Multivariable	4.55 (1.60-12.95)	**0.004**
Median	<5 vs. 5-8	Univariate	0.72 (0.36-1.43)	0.351
		Multivariable	0.75 (0.38-1.49)	0.407
	<5 vs. ≥8	Univariate	0.61 (0.25-1.47)	0.271
		Multivariable	0.67 (0.27-1.64)	0.382
Mean	<5 vs. 5-8	Univariate	0.80 (0.38-1.69)	0.568
		Multivariable	0.84 (0.40-1.78)	0.658
	<5 vs. ≥8	Univariate	0.98 (0.43-2.22)	0.954
		Multivariable	1.05 (0.46-2.40)	0.906

Bold values indicate statistical significance.

### 3.4 Association between TTR and the risk of graft renal insufficiency

**Figure 7:**
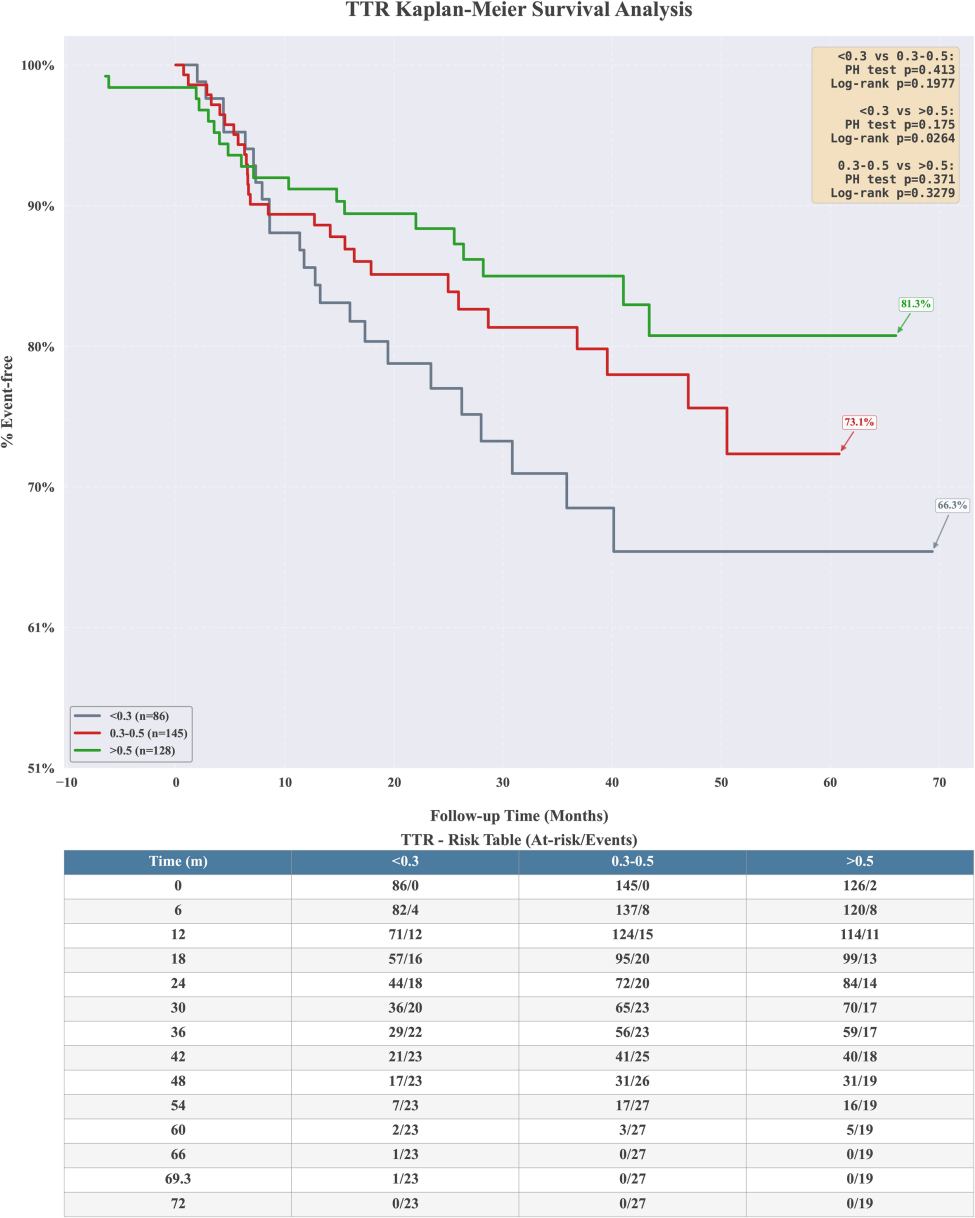
Kaplan-Meier plots of TTR for percent graft renal insufficiency-free survival.

Using different therapeutic range for different time, 59 patients (17.72%) had a TTR ≥50% and 26 (7.81%) patients with TTR ≥70% by 1 months, 156 patients (49.06%) had a TTR ≥50% and 98 (30.82%) patients with TTR ≥70% by 1–3 months, 234 patients (73.35%) had a TTR ≥50% and 172 (53.92%) patients with TTR ≥70% by 3–12 months, and 107 patients (48.64%) had a TTR ≥50% and 59 (26.82%) patients with TTR ≥70% by over 12 months. At all tested thresholds (0.2 to 0.7), the HR values were greater than 1 (ranging from 1.28 to 1.77). This indicated that regardless of the TTR level chosen as the cutoff point, patients with lower TTR consistently exhibited a higher risk of graft dysfunction, consistent with clinical expectations. Unfortunately, none of these associations reached statistical significance. This may be attributed to the limited sample size, which failed to achieve full statistical power. After stratifying patients into three groups based on TTR levels (<30%, 30-50%, >50%), the log-rank test revealed a trend toward significant differences in survival curves between groups (p = 0.026, Bonferroni-corrected). Patients with TTR <30% exhibited the highest cumulative incidence of graft dysfunction. However, no significant differences in survival curves were observed between patients with TTR levels of 30%-50% and those with >50% (**Figure 7**).

## 4 Discussion

To date, the development of novel immunosuppressive agents to improve long-term graft outcomes has been limited, and the optimization of immunosuppression based on immunological risk profiles remains the preferred regimen to improve long-term graft outcomes after transplantation. Identification patients at high-risk of rejection and graft renal insufficiency after transplantation is a significant challenge. The study is of significance as assessed the impact of tacrolimus trough concentration fluctuations on rejection or graft insufficiency following renal transplantation in adults. Several studies have recently explored the impact of TAC variability on adverse outcomes in renal transplantation. However, few studies have focused on the variability of TAC trough levels at different transplantation time. This study hypothesized that fluctuations in TAC trough levels at different times after transplantation would pose a different risk of transplant renal insufficiency. The findings indicated that higher CV of TAC trough levels, higher TAC concentrations and lower TTR were significantly associated with higher risk of graft renal insufficiency.

### 4.1 Effect of the mean, STD and CV of TAC on graft renal insufficiency

When examining the median and mean tacrolimus concentrations at 3–12 months and >12 months post-transplant, no significant dose-response relationship was observed between TAC exposure levels and the risk of graft dysfunction. Only elevated STD values after 12 months post-transplant were associated with a higher risk of graft dysfunction. However, over the entire time period, maintaining TAC concentrations between 5 and 8 ng/mL is beneficial for preserving stable renal function in transplant recipients. Excessively elevated or diminished TAC concentrations during the initial year have been demonstrated to be associated with a progressive deterioration in the risk of graft renal insufficiency over a five-year period. Whilst there is evidence to suggest that minimizing TAC dosage can reduce metabolic, opportunistic infections and nephrotoxicity, this may be accompanied by an increased risk of rejection (8, 19). Conversely, excessive drug exposure has been shown to induce constriction of small glomerular inlet arteries and impaired cortical microcirculation, which in turn leads to an increase in renal vascular resistance, severely affecting long-term graft survival (20).

The CV of TAC trough levels usually refers to the magnitude of fluctuations in whole blood trough concentration measurements over a specific time period (12). In a cohort study of 808 renal transplant recipients, it was found that the higher incidence of the composite end point with a high CV remained statistically significant (21). Another meta-analysis that included 6,638 patients further quantified the effect of CV, with the risk of graft loss increased 32% and 66% when the CV was at 0.3-0.44 and ≥0.45 when compared to patients with a low CV of <30% (19). LinTao et al. developed a new TAC variability score (TVS) by calculating the frequency of clinically significant changes in TAC trough levels after renal transplantation to assess the variability. The study included 1343 patients and used multivariate Cox proportional analyses to compare the effects of TVS and CV on transplantation outcomes. Univariate analysis showed that higher TVS and higher CV were associated with lower graft survival. In multivariate analysis, only TVS was an independent predictor of graft failure (15). In this study, both univariate and multifactorial regression analysis showed that a significant association between CV≥0.5 and the risk of graft renal insufficiency. Persistent fluctuations in TAC concentrations may disrupt immune homeostasis and lead to graft renal impairment by promoting rejection and the chronic inflammatory cascade. Therefore, when abnormal elevations in CV or STD occur, drug adherence, metabolic status, and drug interaction assessment should be performed promptly (22). Recipients with chronically high CV need to be alerted to the potential risk of progressive deterioration of transplant renal function, and frequent monitoring of therapeutic drug concentrations is recommended to optimize individualized dosing regimens.

### 4.2 Effect of the TTR of TAC on graft renal insufficiency

TTR is defined as the percentage of time a patient is within a predetermined target range for a particular medication, and is typically used for warfarin administration (23, 24). It has been posited by other studies that TTR can be utilized for the purpose of blood glucose management monitoring (25). There are two ways of calculating TTR, the time period to reach the recommended range and the number of times the recommended range is reached. To fully evaluate the target range of TAC, we chose to calculate the time required for TAC to reach the target level. The distribution of the TTR shows that the rate is lowest in the early stages, gradually increases in the later stages, and slightly decreases one year after transplantation. In general, only half of recipients achieved TTR≥50%.

However, the results of univariate and multivariate logistic regression demonstrate that, although the TTR did not have a significant effect on graft renal insufficiency, which was different from the CV value. It is hypothesized that this is attributable to the fact that, during the initial six months following transplantation, there is a greater propensity for the utilization of high-dose steroids for the purpose of preventing acute rejection, sulphonamides for the prevention of Pneumocystis carinii, and antivirals for the prevention of cytomegalovirus, among other indications (26). However, the concomitant administration of multiple medications has been demonstrated to affect the absorption or metabolism of TAC, and thus its blood levels. The KM curve analysis, incorporating all TAC concentrations post-transplantation, demonstrated a significant association between lower TTR values and a markedly elevated risk of graft dysfunction. This finding was consistent with the results of recent studies on TAC IPV, which also reported a significant correlation between high IPV and poorer graft function, as well as lower CD4+/CD8+ ratios (27). Cooper et al. evaluated mean TAC trough levels and TAC time in therapeutic range for the risk of *de novo* donor-specific antibodies (dnDSAs). They found that the risk of dnDSA was higher when the mean value of TAC was less than 8ng/mL both by 6 months and 12 months post-transplant, and TAC time in the therapeutic range of <60% was associated with higher risk of dnDSAs (15). Taken together, fluctuations in TAC concentrations not only reflect alterations in drug pharmacokinetics but also serve as an independent risk factor for predicting long-term graft survival, progression of histological damage, and rejection reactions. It is recommended to integrate routine CV and TTR monitoring into electronic medical record systems to establish an active early warning mechanism, thereby enabling early identification and precise intervention for high-risk patients.

### 4.3 Effect of other clinical indicators on graft renal insufficiency

Existing studies suggest that recipient gender may influence transplantation outcomes through multiple mechanisms, but the direction of their effects remains controversial. Several clinical observations have shown that male recipients have a significantly higher risk of early postoperative renal insufficiency, which may be associated with a higher prevalence of smoking and differences in the prevalence of metabolic syndrome in the male population (28, 29). Analysis of the US SRTR database (n=463,895) further revealed a donor-recipient sex interaction that female recipients aged 0–12 years had an increased risk of death with graft function when the donor was male, but no significant difference was observed in other age groups and in female donors (30). Data from our study showed that gender was not significantly associated with transplant renal insufficiency (P = 0.443), we speculated that this may be due to the results of the interaction between age and gender, and that different age groups with different transplantation durations should be used to systematically assess their impact on transplantation outcomes.

According to the findings of Döhler et al. the effect of CV values on transplant renal survival showed a significant age-dependent character. In the group of recipients aged 12–17 years, there was a very strong correlation between an elevated CV value and the risk of graft failure (HR = 4.10, 95% CI 1.97-8.53, P<0.001), whereas in young recipients aged 18–39 years, this risk was reduced but still statistically significant (HR = 1.59, 95% CI 1.12-2.25, P = 0.009) (19). Although the present study reached a different conclusion that no significant effect of age on the prognosis of the grafts. This difference may have stemmed from the peculiarities of the study design. Firstly, the present cohort was strictly limited to adult recipients (≥18 years), and the age distribution showed a clear clustering feature (median age 35 years, IQR 27–43 years), and this relatively narrow age coverage may have weakened the effect of age. Second, the longest follow-up period of 6.8 years (mean 2.5 years) in the present study was significantly shorter than that of more than 10 years in their study. This suggests that age-related biological factors may exist as a time-dependent confounding variable in the assessment of graft prognosis. The strength of its effect is hypothesized to increase with the lengthening of the observation time window, which provides a new perspective for understanding the mechanisms of long-term graft failure. It is postulated that age-related factors may be involved in the progressive impairment of graft function through dynamic processes such as chronic immune senescence or metabolic remodeling.

Cold ischaemia time (CIT) and warm ischaemia time (WIT) are modifiable independent risk factors affecting early injury in transplanted kidneys. Previous international studies have confirmed that the risk of delayed recovery of graft kidney function increased by 23% (OR = 1.23, 95% CI 1.15-1.32) for each 6-hour extension of CIT. In cases where the WIT exceeded 30 minutes, the risk of graft renal loss at one year postoperatively was elevated 1.8-fold (31). The average WIT in our center was controlled at 3.7 minutes, 87% shorter than the national average, and the average CIT was compressed to 3.4 hours, 71% lower than the national median, reaching the international leading level. Our multifactorial analysis showed that neither CIT nor WIT was an independent risk factor for graft renal insufficiency in this cohort (P>0.05), confirming that the risk of graft renal insufficiency can be effectively circumvented through refined surgical management.

## 5 Limitations

The present study identified age and sex maybe have time-dependent cumulative effect on transplanted kidney function. However, given the relatively short follow-up period, the necessity for a long-extended follow-up period in future to elucidating the specific risk. In addition, it was found that lower TTR was associated with higher graft renal insufficiency. However, no subgroup analyses were performed on this population, which has a lower risk of immune damage and a higher risk of infection. This is because lower TAC concentrations may be sufficient for immunosuppression in this population. This study may have been affected by some important confounding factors that influence TAC trough levels and the risk of adverse outcomes. This may have resulted in limitations with regard to the ability to draw conclusions about causality.

Current monitoring based on whole blood trough concentrations has inherent limitations. Even when concentrations remain within the therapeutic window, some patients may experience rejection or adverse reactions, suggesting that concentrations in whole blood may not fully reflect the true pharmacological effects within target cells. Recent studies suggest that monitoring tacrolimus concentrations within peripheral blood mononuclear cells (PBMCs) may be more clinically relevant than traditional whole blood monitoring (32). Therefore, future studies will validate the predictive TTR for long-term outcomes over extended follow-up periods. We will also implement monitoring of tacrolimus concentrations in PBMCs in immunologically unresponsive subgroups to determine the ‘adequate concentration’ threshold for immunosuppression and enable more personalized dosing.

## 6 Conclusion

In conclusion, TAC immunosuppression variability post-transplantation had variable risk of graft renal insufficiency, with greater variability in the early post-transplantation period, but no significant effect on graft renal outcome. Higher TAC trough concentrations, elevated CV values, and lower TTR significantly increase the risk of transplant renal insufficiency, particularly rejection. These findings provide direct evidence for optimizing clinical monitoring strategies. Patients with TTR <0.3 or CV >0.5 post-transplantation should be flagged as high-risk and subjected to more intensive concentration monitoring and follow-up. Identifying these high-risk patients and correcting any factors that may be contributing to unstable levels may significantly improve their outcomes and survival.

## Data Availability

The raw data supporting the conclusions of this article will be made available by the authors, without undue reservation.
