# Supplementary material for: Elevated tacrolimus exposure variability predicts transplant renal insufficiency
Source: Front Immunol. 2026 Feb 13;17:1656392. doi: 10.3389/fimmu.2026.1656392 (PMC12945822; doi:10.3389/fimmu.2026.1656392)
Supplement: Supplementary file 1 [file DataSheet1.docx]

**Elevated Tacrolimus Exposure Variability Predicts** **Transplant Renal Insufficiency**

Yue Zhao^1, †^, Ying-Xin Zhao^2, †^, Di Zhao^1^, Wei-Li Wang^3, *^, Qian Wang^1, *^

^1^Department of Pharmacy, the First Affiliated Hospital of Army Medical University (Third Military Medical University), Chongqing 400038, P.R. China

^2^Department of Pharmacy, Army Medical Center, Army Medical University (Third Military Medical University), Chongqing 400042, P. R. China

^3^ Department of Nephrology, the First Affiliated Hospital of Army Medical University (Third Military Medical University), Chongqing 400038, PR China

Corresponding information:

**Corresponding author name: Wei-Li Wang**

Institution: Department of Nephrology, the First Affiliated Hospital of Army Medical University, Chongqing 400038, P. R. China

E-mail: cqwangweili@tmmu.edu.cn

**Corresponding author name: Qian Wang**

Institution: Department of Pharmacy, the First Affiliated Hospital of Army Medical University, Chongqing 400038, P. R. China

E-mail: wangqian411@tmmu.edu.cn

The first two authors equally contribute to this paper.

Figure S1. The STD vs Median TAC level at various times after transplantation

Figure S2. Hazard ratio for graft renal insufficiency using a binary cutoff of ≥ or < mean, median, STD and CV of TAC level at 3-12 months

Figure S3. Hazard ratios of graft renal insufficiency by TAC level metrics (mean, median, STD, CV) at 3-12 months


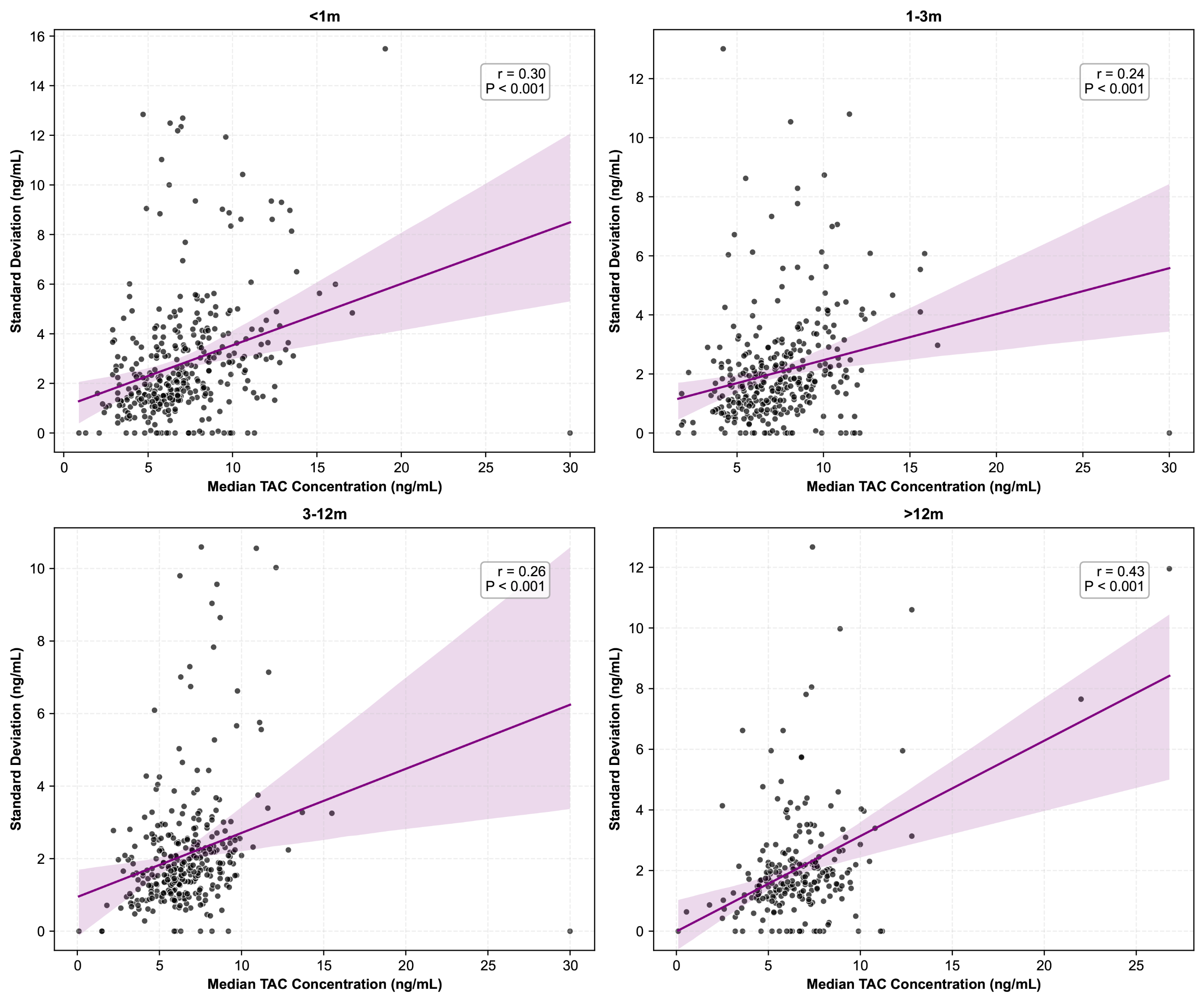


Figure S1. The STD vs Median TAC level at various times after transplantation


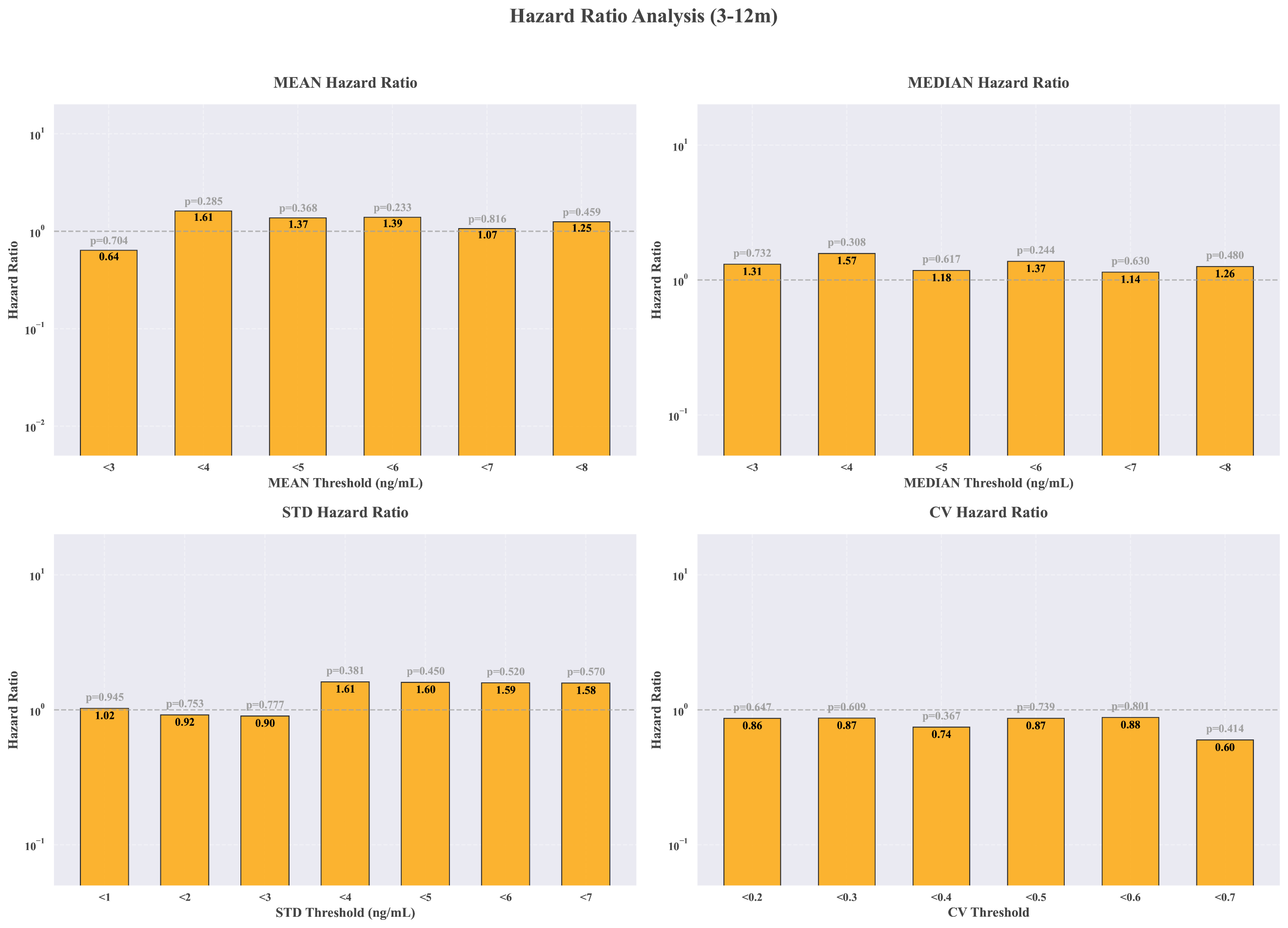


Figure S2. Hazard ratio for graft renal insufficiency using a binary cutoff of ≥ or < mean, median, STD and CV of TAC level at 3-12 months


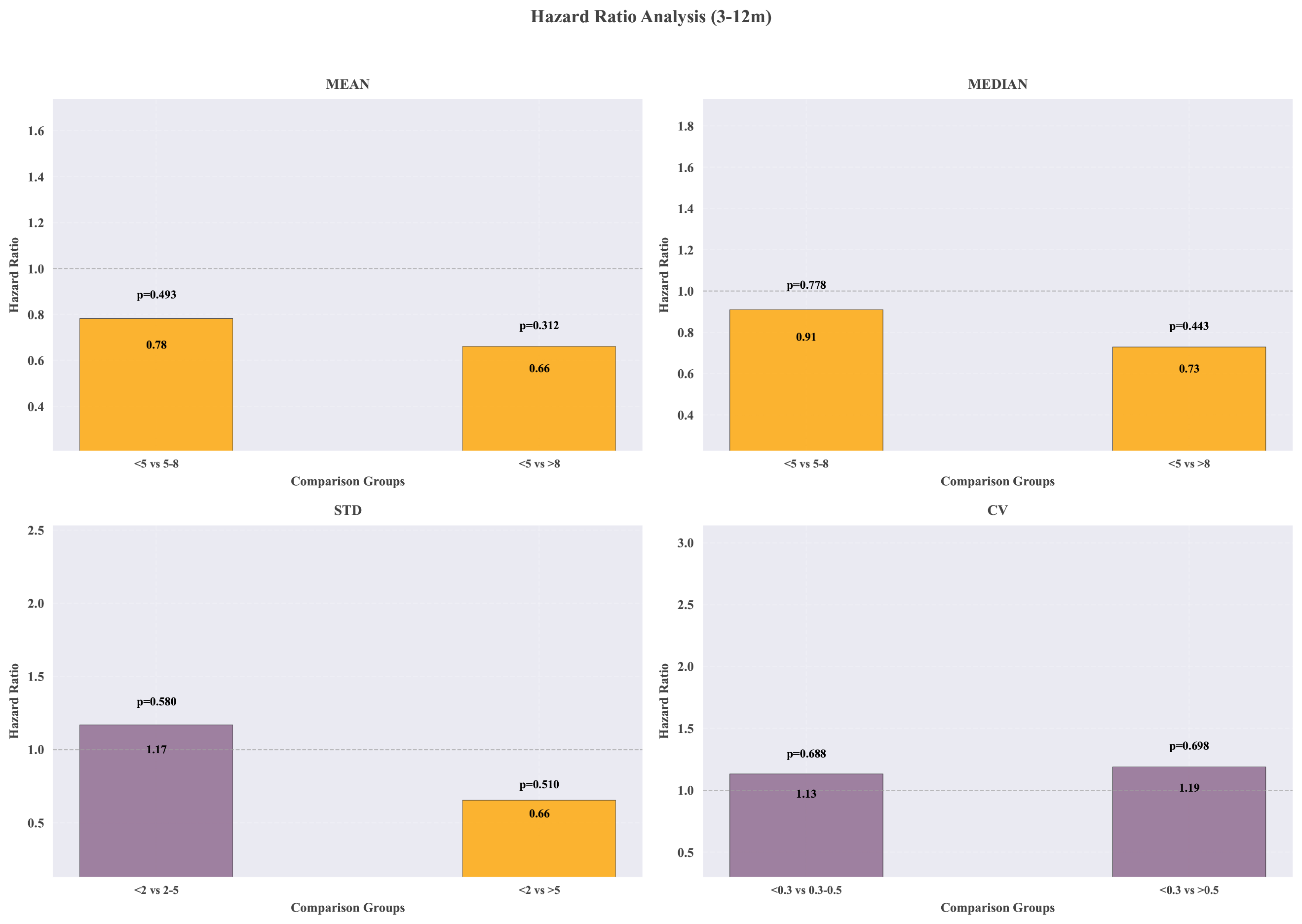


Figure S3. Hazard ratios of graft renal insufficiency by TAC level metrics (mean, median, STD, CV) at 3-12 months
